# Bronchoscopy for foreign body aspiration and effects of nebulized albuterol and budesonide combination

**DOI:** 10.12669/pjms.331.11297

**Published:** 2017

**Authors:** Bulent Akcora, Mehmet Emin Celikkaya, Cahit Ozer

**Affiliations:** 1 Bulent Akcora, MD, Associate Professor, School of Medicine, Department of Pediatric Surgery, Mustafa Kemal University, 31124, Antakya, Hatay, Turkey; 2 Mehmet Emin Celikkaya, MD, School of Medicine, Department of Pediatric Surgery, Mustafa Kemal University, 31124, Antakya, Hatay, Turkey; 3 Cahit Ozer, MD, Professor, School of Medicine, Department of Family Medicine, Mustafa Kemal University, 31124, Antakya, Hatay, Turkey

**Keywords:** Albuterol, Aspiration, Budesonide, Foreign body

## Abstract

**Objective:**

A foreign body aspiration in the tracheobronchial tree is a dangerous medical condition in the childhood period. Although rigid bronchoscopy is a safe procedure, it may cause complications. The aim of this study was to present our bronchoscopy experience and to evaluate the efficacy of pre-operative administration of nebulized albuterol and budesonide combination for reducing intra-operative complications in foreign body aspirated cases.

**Methods:**

In this retrospective study our pediatric cases in which a foreign body was removed from tracheobronchial tree in last 8 years were analyzed. After excluding the patients who needed emergent and negative bronchoscopies, the remaining clinically stable 84 patients were compared for the effects of preoperative administration of nebulized albuterol and budesonide combination on bronchoscopy complications.

**Results:**

There were 51 boys (60.3%) and 33 girls (39.7%). There were 38 children in the non-nebulized group and 46 children in the nebulized group. We found that the combined albuterol and budesonide nebulization decrease complications such as arterial oxygen desaturation (p<0.05), and bronchospasm (p<0.05) during the bronchoscopic intervention.

**Conclusion:**

Preoperative nebulization of albuterol and budesonide combination may decrease perioperative complications of bronchoscopy.

## INTRODUCTION

Tracheobronchial foreign body aspiration (FBA) is a common and life-threatening problem among children. FBA may cause complete occlusion of airways and sudden death.^1^,^2^ It is the most important cause of accidental death of children under six years of age.^3^ In addition; the aspirated foreign bodies (FB) that reach to the distal segments of tracheobronchial tree can cause serious complications such as recurrent bronchopneumonia, atelectasis, and bronchiectasis.^1^,^2^ Early diagnosis and bronchoscopic extraction of aspirated objects are essential to prevent morbidity and mortality.^4^

Rigid bronchoscopy is a very safe and effective way for removal of FB. However, perioperative complications or mortality can occur during this procedure. There are many factors such as size and location of FB, pre-operative management, method of anesthesia, the general condition of the child, the elapsed time after aspiration and the experience of the surgeon which affect the final outcome.^4^

The aim of this retrospective study was to present our bronchoscopic FB removal experience and to evaluate whether pre-operative administration of nebulized albuterol(bronchodilator) and budesonide (corticosteroid) combination for reducing intra-operative complications in FBA cases.

## METHODS

The medical records of children underwent bronchoscopy for FBA at the Department of Pediatric Surgery of Mustafa Kemal University between January 2009 and June 2016 was analyzed retrospectively with Institutional Ethics Committee Approval (16/06/2016/163).

Patients with FB aspiration and clinically stable conditions (n=84) which underwent elective bronchoscopy for FB removal were included in the study. Exclusion criteria were patients who needed emergent bronchoscopies and those who had negative bronchoscopies.

Our department’s routine approach to children of suspected FBA included the physical examination and posteroanterior chest x-ray. If an emergent endoscopy is needed for children with severe respiratory distress, the patient is referred immediately to operating room. However, children were relatively stable generally. Clinically stable cases were hospitalized for detailed physical examination, pre-anesthetic evaluation, intravenous hydration, antibiotic treatment if necessary, sufficient fasting time for safe anesthesia or daytime operation. Since July 2013, we had been using a combination of nebulized albuterol (Ventolin, Glaxo Smith Kline) in dose of 0.15 mg/kg every 6 hours and budesonide (Pulmicort, AstraZeneca) in dose of 0.015 mg/kg every 12 hours combination to clinically stable children before bronchoscopy for decrease irritative and inflammatory effects of FB to the tracheobronchial tree. This protocol was not in use before June 2013 in our patients.

All bronchoscopies were performed in the operating room under general anesthesia with myorelaxant using ventilating type pediatric bronchoscope (Karl Storz) of appropriate size for child’s weight and age. Electrocardiogram, heart rate, oxygen saturation (with pulse oximetry) and non-invasive blood pressure were obtained from all patients during the procedure. After tracheobronchial system examination, the FB was removed using optical forceps (10378KF or 10378L, Karl Storz). The telescope was re-inserted to evaluate for remaining fragments, mucosal inflammation and aspiration of secretions in the tracheobronchial tree after extraction of the FB. Then mechanical ventilation was continued with endotracheal intubation or mask until the return of spontaneous breathing. All patients received 1 mg/kg prednisolone at the end of bronchoscopy.

SPSS 13 software was used for statistical analyses. Data was presented as frequencies and percent or median with minimum and maximum. Chi-square or Fisher’s exact test was used to compare categorical variables between the groups. Mann-Whitney U test was used to compare continuous variables between groups. P level was accepted as 0.05.

## RESULTS

A total of 84 cases were included in this study. There were 51 boys (60.3%) and 33 girls (39.7%). Their ages ranged from 6 months to 15 years with a median age of 3.5 years. The majority of the patients (75%, n=63) were less than three years old. The duration between aspiration and hospital admission varied from one hour to two months (median=21 hours).

Presenting complaints were cough (91.6%, n=77) choking episode (35.7%, n=30) and noisy breathing (25.0%, n=21). Decreased breath sounds and wheezing were the most prominent findings of physical examination. The clinical signs of patients are presented in Table-I.

**Table-I:** Clinical findings.

Clinical Signs		

	No. Patients	Percent (%)
Cough	72	85.7%
Decreased breath sound	45	53.5%
Wheezing	24	28.5%
Normal	11	13.1%
Ralles	10	11.9%
Tachypnea	7	8.3%
Stridor	2	2.3%

Aspirated objects were removed from the following locations; right bronchial tree (44%, n=37), left bronchial tree (33.3%, n=28), trachea (19%, n=16) and bilateral bronchial trees (3.6%, n=3). The most common type of FB (85.7%, n=72) was organic materials such as dried nuts and sunflower/melon seeds. The other foreign bodies removed from the tracheobronchial tree were shown in Table-II.

**Table II:** Type of foreign body.

	No. Patients	Percent (%)
Dried nuts	37	44.0%
Sunflower or Melon Seeds	25	29.8%
Needle	6	7.1%
Nonspecific foods	6	7.1%
Carrot	3	3.6%
Toy peace	2	2.3%
Stone	1	1.2%
Teeth	1	1.2%
Bone fragmant	1	1.2%
Pen cap	1	1.2%
Lightbulb	1	1.2%

Total	84	100%

The duration between hospitalization and bronchoscopy ranged between 2 to 48 hours in all patients. Observed peri-interventional complications and general data such as heart rate, saturation and durations were documented in Table-III for non-nebulized 38 patients and nebulized 46 patients. We found that desaturation and bronchospasm decreased in the nebulized group significantly (p<0.05) comparing to the non-nebulized group. There were no statistically significant difference between the two group regarding the laryngeal edema, bleeding and bradycardia.

**Table III:** Complications and General Data.

	Non-nebulized patients (n= 38)	Nebulized patients (n= 46)	P
*Preoperative*			
Median (min-max) duration between hospitalization and bronchoscopy (hour)	5.5 [2-48]	12 [2-35]	0.566
*Peroperative*			
Desaturation (no. patients)	6	1	0.043
Bronchial spasm (no. patients)	4	0	0.038
Severe laryngeal edema (no. patients)	2	1	0.587
Bradycardia (no. patients)	0	1	0.548
Bleeding (no. patients)	0	1	0.558
Median (min-max) arterial saturation (%)	99 [85-100]	99 [83-100]	0.133
Median (min-max) heart rate (beats/min)	127 [90-185]	140 [80-170]	0.250
Median (min-max) duration of anestesia (second)	15 [4-65]	15 [5-50]	0.455
Median (min-max) duration of bronchoscopy (second)	35 [20-100]	30 (15-65]	0.872
*Postoperative*			
Pneumonia (no. patients)	2	1	0.587
Median (min-max) duration of hospital stay after procedure (hour)	22 [3-220]	15 [2-65]	0.090

## DISCUSSION

Anamnesis for suspicion of FBA into the respiratory tract is the most important part of the diagnosis. Presenting symptoms known as ‘‘penetration syndrome’’ includes sudden onset of choking crisis and paroxysmal cough with or without emesis is generally seen.^5^ Similarly, choking crisis during nourishment and cough were the most important complaints of parents in our cases.

FBA may cause highly variable physical examination findings related to the size and location of inhaled objects. If FB leads to significant obstruction of the trachea or both of the main bronchi, symptoms of severe respiratory distress such as stridor, cyanosis and tachypnea, even sudden death may be seen.^6^,^7^ Urgent bronchoscopic removal of FB is mondatory in children with signs of severe respiratory distress. However, generally the clinical situation is relatively stable. When FB locates at more distal airways, symptoms may be minimal.^6^ Mani et al. reported that waiting (mean 12.6 hours) to the next working day for bronchoscopy under optimal condition does not increase the morbidity or mortality in clinically stabile patients.^8^

If aspirated FB is organic nature, it may cause significant mucosal inflammation, edema and granulation tissue.^6^ According to our opinion these effects of FB may cause irritation and bronchospasm on airways which show itself with cough and wheezy in physical examination. In our series, the most common type of FB (86.2%) were organic materials such as dried nuts and sunflower/melon seeds relevant with the result of other studies. In addition, cough and wheezing were important clinical signs.

FBA may cause a prompt breathing insufficiency, chronic and irreversible pulmonary damage, or death. Although some deaths occur before reaching the hospital, removing of aspirated bodies with bronchoscopy may cause some complications such as bronchospasm, desaturation and laryngeal edema. The complication rate of rigid bronchoscopy for FB extraction was reported to be between 3% and 29%.^1^,^9^-^11^ Bronchospasm and desaturation are the most common complications of this procedure. If it isn’t treated promptly, it can cause hypoxic cardiac arrest and death.^12^ Laryngospasm is another serious complication that is seen approximately in 10% of patients with upper airway interventions.^13^

Bronchoscopists use some medications such as intravenous corticosteroid (prednisolone) or atropine sulfate^14^ preoperatively to decrease secretions and edema or to prevents vasovagal reflex during bronchoscopy.^15^ Zhijun et al. reported that topical anesthesia of respiratory tract during endoscopy with tetracaine or lidocaine to avoid laryngospasm is very effective.^4^ Mehta et al. demonstrated that post-bronchoscopic administration of the nebulized albuterol, budesonide and lidocaine combination decrease some complications such as tracheobronchial spasm, edema and inflammation in children with FBA.^16^

Albuterol (β2 adrenergic receptor agonist) and budesonide have an important place in the relief of acute and chronic pulmonary problems in children. Albuterol is used for acute, preventative or maintenance therapy for bronchospasm in asthmatic patients. Budesonide is an important anti-inflammatory agent and there is much evidence showing that it provides a successful relief of asthmatic symptoms in many patients either alone or in combination with bronchodilators.^17^

Payne et al. has shown that using of preoperative corticosteroid decrease bronchoscopic complications such as laryngospasm, desaturation and apnea in asthmatic children.^18^ Similarly, nebulization of β2 adrenergic receptor agonist suggested for decreasing bronchospasm during flexible bronchoscopy in patients with asthma and bronchial hyperactivity.^19^

There is no consensus about using short acting bronchodilator before bronchoscopy toreduce complications in adult patients with chronic obstructive pulmonary disease (COPD).^20^ Hattotuwa et al. has suggested inhalation of salbutamol for ensuring safety of bronchoscopy with purposes of bronchoalveolar lavage and biopsy in patients with COPD. 21 A randomized, placebo controlled trial has not recommended premedication with β2 adrenergic receptor agonist in patients with COPD undergoing bronchoscopy because it does not improve safety.^20^

Since July 2013, we have been using nebulized albuterol and budesonide combination to clinically stable children before bronchoscopy. We had two objectives of using this treatment. The first was to decrease irritative or inflammatory effects of FB to the tracheobronchial tree and relieve some symptoms including an intractable cough and wheezing until bronchoscopy. The second aim was to decrease operative complication such as desaturation, bronchospasm and laryngeal edema during bronchoscopy. Albuterol (0.15 mg/kg every 6 hours) and budesonide (0.015 mg/kg every 12 hours) treatment was started immediately after hospitalization of child and continued until bronchoscopy. Median duration of preoperative treatment was 12 [2-35] hours in treatment group. Twenty six (30.9%) patients were operated within the first 6 hours. So, they only took one dose of medication before bronchoscopy.

In our retrospective study, there were 38 children in the non-nebulized group and 46 children in the nebulized group. When compared with each other, we found that albuterol and budesonide nebulization significantly decreased on bronchoscopic complication such as desaturation, and bronchospasm during bronchoscopy. We feel that administration of this combination might have decreased the irritative, bronchospastic effects of both FB and bronchoscope on tracheobronchial tree. In terms of laryngeal edema, bradycardia and bleeding the effect of treatment was not different significantly between two groups.

Interestingly, two patients in the nebulized group expelled the FB (corn and peanut) spontaneously while coughing. Both of them were admitted to the hospital in the first 6 hours after aspiration and both had clinical and radiological signs indicating bronchial FB. Bronchoscopy was done to rule out any residual peace after spontaneous expectoration of FB but could not find anything. In the past, Cotton et al. used bronchodilator and postural drainage (nonendoscopic technic) for expectorate of aspirated FB in 24 child succesfully.^22^ Sabra et al. reported that albuterol inhalation may cause substitution of FB and potential danger of suffocation,^23^ but we did not encounter such a problem in our patients.

Rigid bronchoscopy is the most effective way in the management of FB aspiration. Preoperative nebulization with albuterol and budesonide may decrease perioperative complications and may cause spontaneous expectoration of FB. However, further prospective, randomized clinical studies showing the effects of these agents on the bronchoscopic treatment of FBA are needed.
